# *Centella Asiatica* Improves Memory and Promotes Antioxidative Signaling in 5XFAD Mice

**DOI:** 10.3390/antiox8120630

**Published:** 2019-12-08

**Authors:** Donald G Matthews, Maya Caruso, Charles F Murchison, Jennifer Y Zhu, Kirsten M Wright, Christopher J Harris, Nora E Gray, Joseph F Quinn, Amala Soumyanath

**Affiliations:** 1 Department of Neurology, Oregon Health and Science University, Portland, OR 97239, USA; matthedo@ohsu.edu (D.G.M.); carusma@ohsu.edu (M.C.); cfmurch@uab.edu (C.F.M.); zhje@ohsu.edu (J.Y.Z.); wrigkir@ohsu.edu (K.M.W.); harrichr@ohsu.edu (C.J.H.); grayn@ohsu.edu (N.E.G.); quinnj@ohsu.edu (J.F.Q.); 2 Department of Biostatistics, University of Alabama at Birmingham, Birmingham, AL 35294, USA; 3 Parkinson’s Disease Research Education and Clinical Care Center, Veterans’ Administration Portland Health Care System, Portland, OR 97239, USA

**Keywords:** *Centella asiatica*, Alzheimer’s disease, 5XFAD, mouse model, oxidative stress, antioxidant, NRF2, neuritic dystrophy, cognitive function, memory

## Abstract

*Centella asiatica* (CA) herb is a traditional medicine, long reputed to provide cognitive benefits. We have reported that CA water extract (CAW) treatment improves cognitive function of aged Alzheimer’s disease (AD) model Tg2576 and wild-type (WT) mice, and induces an NRF2-regulated antioxidant response in aged WT mice. Here, CAW was administered to AD model 5XFAD female and male mice and WT littermates (age: 7.6 +/− 0.6 months), and object recall and contextual fear memory were tested after three weeks treatment. CAW’s impact on amyloid-β plaque burden, and markers of neuronal oxidative stress and synaptic density, was assessed after five weeks treatment. CAW antioxidant activity was evaluated via nuclear transcription factor (erythroid-derived 2)-like 2 (NRF2) and NRF2-regulated antioxidant response element gene expression. Memory improvement in both genders and genotypes was associated with dose-dependent CAW treatment without affecting plaque burden, and marginally increased synaptic density markers in the hippocampus and prefrontal cortex. CAW treatment increased *Nrf2* in hippocampus and other NRF2 targets (heme oxygenase-1, NAD(P)H quinone dehydrogenase 1, glutamate-cysteine ligase catalytic subunit). Reduced plaque-associated SOD1, an indicator of oxidative stress, was observed in the hippocampi and cortices of CAW-treated 5XFAD mice. We postulate that CAW treatment leads to reduced oxidative stress, contributing to improved neuronal health and cognition.

## 1. Introduction

*Centella asiatica* (CA) has been utilized for its cognitive benefits for centuries in traditional Chinese and Ayurvedic medicine. Modern scientific studies in rodents [1,2,3] and in human subjects [4,5] have shown cognitive-enhancing or neurotropic properties of whole CA extracts, as well as some of its known active components [6]. These properties of CA may have relevance for the treatment of Alzheimer’s disease (AD).

Treatment with CA in rat models improves cognitive function and shows neuroprotection in chemically induced, AD-like memory loss [7]. In addition, CA-derived treatments mitigate oxidative stress [8,9,10] and mitochondrial dysfunction in rodents [11]. A CA ethanol extract (2500 mg/kg/d for eight weeks) decreases Aβ levels in the PSAPP AD mouse model, which has a mutation in both the amyloid precursor protein (APP) and presenilin 1 (PS1) proteins [12]. By contrast, our laboratory reported the cognitive benefits of a CA water extract (CAW; 200 mg/kg/day for five weeks) in females from the Tg2576 APP mouse model of AD [13], but, notably, without an impact on Aβ levels. This led us to examine mechanisms downstream of Aβ deposition as mediators of CAW’s effects.

APP mouse models of AD demonstrate cognitive decline with pathology, comprising Aβ plaques closely associated with areas of high oxidative stress and dystrophic neurites [14,15]. Aβ plaques also play a role in facilitating a positive feedback loop of increasing oxidative stress and subsequently elevated Aβ levels, leading to neuritic dystrophy and culminating in neuronal death [16,17,18,19]. Oxidative damage is particularly relevant to the brain, due to its high polyunsaturated fatty acid content and oxygen consumption rate, even under ideal brain conditions. Mitigation of oxidative damage caused by reactive oxygen species (ROS) is integral to neuronal health, particularly in opposition to AD pathology. Superoxide dismutase (SOD) enzymes are strong antioxidants that play a key role in the mediation of oxidative damage via the removal of excess ROS. Unsurprisingly, increased SOD expression is associated with dystrophic neurites adjacent to Aβ plaques in immunohistochemistry (IHC) analysis of AD models [20,21]. 

In addition to several antioxidant enzymes (e.g., SOD, catalase, and glutathione peroxidase), ROS levels can be reduced by activation of the nuclear factor (erythroid-derived 2)-like 2 (NFE2L2; NRF2) pathway, and, subsequently, the antioxidant response element (ARE) genes. NRF2 alleviates oxidative stress, by regulating the transcription of the target ARE genes heme oxygenase 1 (*Ho-1*), NAD(P)H dehydrogenase quinone 1 (*Nqo1*) and glutamate–cysteine ligase catalytic subunit (*Gclc*). *Ho-1* and NQO1 are upregulated in AD patients [22,23], while GCLC is the main regulating protein for glutathione production, a process impacted by AD pathology [24,25,26]. 

Our laboratory and others have investigated the mechanisms involved in CAW’s neuroprotective effects in vitro. CAW increases NRF2-regulated gene transcripts, reduces Aβ-associated ROS [27], and increases spine density and dendritic arborization in Tg2576 primary hippocampal neurons [28]. Also, CA extracts have demonstrated positive effects on neurite elongation [6], neurodegeneration [29], antioxidant activity [30], and mitochondrial dysfunction [31]. These observations are the subject of a recent review [32].

We have confirmed that these mechanisms also operate in aging wild-type (WT) mice [1]. In this study, we expand on our previous studies in female Tg2576 [13] and female 5XFAD mice [33] treated with 200 mg/kg/d CAW. The present study includes 5XFAD mice and WT littermates of both genders and treatment with a range of CAW doses (0, 200, 500, 1000 mg/kg/d). The 5XFAD model of AD allows for an accelerated timetable of cognitive dysfunction associated with Alzheimer’s Aβ pathology compared to the Tg2576 model, in addition to modeling the hippocampal neuron loss indicative of AD, which is absent from the Tg2576 model [34,35]. 5XFAD mice accumulate detectable Aβ_42_ within neurons by six weeks of age, and extracellular deposits of Aβ by eight weeks, leading to ubiquitous plaque formation in the hippocampus and cortex at six months, coinciding with the start of neuronal loss [35,36]. Significant memory impairment is observed before six months of age [35,37,38], months before pathology and cognitive deficiencies develop in Tg2576. This practical consideration enables expanded experimentation, including an increased number of cohorts treated with a range of CAW doses and analysis of both genders. In the original paper characterizing Tg6799 (5XFAD) pathology [35], female mice demonstrated increased levels of Aβ_42_ and Aβ_40_ compared to males. Bundy et al. showed that this may be due to increased expression of the transgene in female 5XFAD mice [39]. Females also appear to have increased BACE1 activity, a β-secretase processing enzyme which cleaves pAPP and produces pathogenic Aβ fragments [40,41], ultimately leading to increased amyloid plaque burden compared to male 5XFAD mice [41,42]. Increased plaque density in females corresponds with a more robust inflammatory response than in males [43], but does not necessarily manifest in poorer learning and memory [44], or convey a gender-mediated change in life expectancy [45]. Here we utilize both female and male, 7–9 month old 5XFAD mice and WT littermates to investigate possible gender differences in the cognitive benefits of CAW treatment and its associated mechanisms. 

In this study, both male and female 5XFAD mice recapitulated CAW’s cognitive benefit in a dose-responsive manner without impacting Aβ plaque formation. In addition, CAW positively impacted antioxidant and synaptic gene expression in the hippocampus (and, to a lesser extent, in the frontal cortex) over untreated levels regardless of pathology. The observed CAW effects were predominantly gender-independent, suggesting a potential benefit to both men and women in upcoming clinical trials.

## 2. Materials and Methods 

### 2.1. CAW Production and Administration

*Centella asiatica* herb, consisting of dried aerial parts, was purchased from Oregon’s Wild Harvest (Redmond, OR, Lot # 170300206). *Centella asiatica* dried water extract (CAW) was prepared by separately boiling, under reflux, several batches of dried herb (160 g) in distilled water (2 L) for two hours. While still hot, extracts were filtered through Whatman filter paper (185 mm; Grade 1) to remove plant debris, and the filtrate cooled, and lyophilized to yield a dried material (CAW). Dried extracts were stored at −20 °C until use. Voucher samples of the original dried plant material have been deposited at Oregon State University Herbarium (OSC-V-258629), while voucher samples of plant material and the extracts (CAW α-θ) are held in our laboratory. Plant identity was verified at the suppliers by visual inspection and Fourier transform Infra-Red spectroscopy (Model: Nicolet 380, ThermoElectron North America LLC, Division of ThermoFisher, West Palm Beach, FL, USA), and in our laboratory with the use of thin layer chromatography to compare with earlier lots [13,46] and the literature [47]. CAW (0, 2, 5, or 10 g/L) was provided to animals ad libitum in their water bottles. Based on average daily decrease in water volume in the bottles, the range of mouse body weights, and varying number of mice per cage, we calculated respective dosing ranges to be 0, 177–457, 442–1143, and 884–2285 mg of CAW per kg of body weight per day (mg/kg/d). However, taking into account that not all CAW water used was actually consumed, and that additional water losses may be due to normal mouse activity and the dispensing mechanism of the water bottle, we estimated towards the lower end of these ranges and denote these treatments as 0 (controls), 200, 500, or 1000 mg of CAW per kg of body weight daily.

### 2.2. Animals 

All procedures were conducted in accordance with the NIH Guidelines for the Care and Use of Laboratory Animals and were approved by the Institutional Animal Care and Use Committee of the Portland VA Medical Center (IACUC #: 3260-17). 5XFAD male mice were bred with a female of the hybrid background C57BL/6:SJL F1 purchased from Jackson Laboratory (Bar Harbor, ME, USA). 5XFAD progeny and WT littermates were identified by PCR of transgenic hAPP from DNA tail samples. Animals were housed in a climate-controlled environment with an alternating 12 h light–dark schedule and fed PicoLab Laboratory Rodent Diet 5L0D (LabDiet, St. Louis, MO, USA). Food and water were given ad libitum. Male and female 5XFAD and WT littermates (7.6 +/− 0.6 months) were treated for five weeks with or without CAW (0, 200, 500, 1000 mg/kg/d) in the drinking water, for a total of 16 groups and behavioral testing performed during weeks three and four of treatment. Animals were sacrificed after five weeks of treatment (nine months of age) and tissues were harvested and fixed as appropriate. Brain was dissected at sacrifice into the following parts, and snap frozen: prefrontal cortex (2–3 mm of anterior frontal lobes), cerebellum, brain stem, left hippocampus, left cortex, and left deep grey. The intact right hemisphere (minus prefrontal cortex, cerebellum, and brain stem) was processed as described under immunohistochemistry.

### 2.3. Novel Object Recognition Task (NORT)

Open Field (Days 1–2): Each mouse was placed in a square arena (38 × 38 × 64 cm high, constructed of white acrylonitrile butadiene styrene) for 10 min each day and allowed to freely explore the area for environmental acclimatization. The arena was wiped down with 70% ethanol in between each mouse trial to eliminate scent cues and clean and disinfect the area. Object Exploration (Day 3): Mice were allowed to familiarize themselves with two identical objects. During training, two identical objects, A1 and A2, were placed into the NW and SW corners of the arena 10 cm away from the north and west walls, and south and west walls, respectively. Objects were composed of non-porous material plastic, metal, or glass for ease of sanitation and were between the dimensions of 6 cm (length) × 6 cm (width) x 10 cm (height). Mice were video recorded using video tracking software (Any-Maze Software version 6, Stoelting Co., Wood Dale, IL, USA). Mice participated in 10 min training trials three times, separated by one hour. In between each trial, the arenas and objects were sprayed down with 70% ethanol to eliminate scent cues. Object Recognition (Day 3–4): 2 h and 24 h after the third training trial, one object was replaced with a previously unexplored object in the identical position to the replaced object and mice were tested for 10 min. At the 24 h retention trial, mice were given a second novel object, replacing the novel object from the 2 h trial. During the 2 h and 24 h trials, mice were video recorded using Any-Maze Software. In between each trial, the arena and objects were sprayed down with 70% ethanol to eliminate scent cues. All video was scored by a technician blinded to the experimental condition to determine when and how long a mouse was exploring each object. Exploring an object was defined as having the nose or front paw within 2 cm of the object and not climbing on the object. Mice were tested on their preference for exploring the novel object (i.e., their memory retention of the familiar object), illustrated as a percentage of time spent exploring the novel object divided by the total time exploring all objects. Mice failing to participate in the study (i.e., explore either object for a period of 12 s) were excluded from analysis.

### 2.4. Conditioned Fear Response (CFR)

Using our conditioned fear paradigm, mice were trained to associate the testing cage with an unpleasant experience while a freezing behavior was assessed. Mice were allowed a habituation period of five minutes as a measure of baseline freezing. Immediately after this, three consecutive one minute periods were observed, in which a mild shock (1 s, 0.5 mA) was administered to their feet at a random time within each of the one minute intervals. The randomness of the shock stimulus was regulated by Fusion v6.0 for SuperFlex Edition Software (Omnitech Electronics, Inc, Columbus, OH, USA). After a 24 h resting phase, each mouse was reintroduced to the testing environment and given a freezing score via AnyMaze Software (version 6, Stoelting Co., Wood Dale, IL, USA) over a 5 min contextual fear testing interval. No shock was administered during this contextual fear testing phase. Freezing time during contextual fear testing was corrected for baseline freezing during the habituation period, and expressed as a percentage of the 5 min observation period. All freezing was measured in real time using AnyMaze tracking software (version 6, Stoelting Co., Wood Dale, IL, USA).

### 2.5. Quantitative PCR (qPCR)

Total RNA was harvested from mouse left hippocampal and left and right prefrontal cortex tissue via Tri-Reagent extraction (Molecular Research Center, Cincinnati, OH, USA) and cDNA produced via reverse transcription using the Superscript III First Strand Synthesis Kit (Invitrogen, Carlsbad, CA, USA) per manufacturers’ instructions. Relative gene expression was measured using Taqman primers and probes (nuclear factor (erythroid-derived 2)-like 2 (*Nfe2l2*; *Nrf2* - Mm00477784_m1), NAD(P)H dehydrogenase-quinone oxidoreductase 1 (*Nqo1*; Mm001253561_m1), glutamate-cysteine ligase, catalytic subunit (*Gclc*; Mm00802655_m1), heme oxygenase 1 (*Ho-1*; Mm00516005_m1), post-synaptic density protein 95 (*Psd95*; Mm00492193_m1)) and reagents (TaqMan Gene Expression Master Mix) from Applied Biosystems (Foster City, CA, USA) with normalization to glyceraldehyde-3-phosphate dehydrogenase (*Gapdh*; hs02758991_g1 (Applied Biosystems, Foster City, CA, USA)) expression. qPCR was performed on a StepOne Plus Machine (Applied Biosystems, Foster City, CA, USA) and analyzed using the delta-delta Ct method. All groups were normalized to untreated WT females for evaluation.

### 2.6. Immunohistochemistry

Right hemispheres were dissected at sacrifice, fixed in 4% formaldehyde/PBS and transferred to progressively concentrated sucrose solutions from 0% to 30% sucrose, and stored at −80°C until sectioning for IHC. Samples were coronally sectioned at 40 microns on a cryo-histomat. Similar depth sections were incubated in a quenching solution (30% methanol, 0.3% H_2_O_2_ in 1× TBS) for endogenous catalase activity and blocked (10% horse serum, 2% BSA, 0.5% triton in 1× TBS) in 6-well plate Netwell inserts. Sections were subsequently incubated with (1:1000) pan Aβ antibody (44–136, Invitrogen, Carlsbad, CA, USA) or SOD1 (PC077, The Binding Site, Birmingham UK) overnight at room temperature. Biotin-labeled anti-rabbit (pan Aβ or anti-sheep (SOD1) secondary antibody (1:200) (Vector laboratories, Burlingame, CA, USA) was used to label the sections and ABC kit (Vector laboratories, Burlingame, CA) was used to visualize staining. DAB (Sigma Fast 3,3 Diaminobenzidine Tablet Set, D-4418 (Sigma-Aldrich Corp, St. Louis, MO, USA)) staining was used as a counterstain. Sections were then mounted on slides and scanned using PrimeHisto XE (Pacific Image Electronics, Torrance, CA, USA). To quantify staining using ImageJ software (version 2 (FIJI), Johannes Schindelin, Max Planck Institute of Molecular Cell Biology and Genetics, Dresden, Germany), images were converted to 8-bit grayscale, and the polygon tool was used to encompass the hippocampus or cortex and the total area of each brain area was recorded. The threshold was adjusted to remove ubiquitous background staining, highlighting only areas of intense staining. “Particles” were counted as the number of individual areas of staining (foci) that were higher than the threshold value. This threshold value was applied across all 5XFAD sections within that particular set of stains and was repeated for each subsequent set of stains, each including a WT littermate brain and human AD brain used as a negative and positive control, respectively. Three sections from each brain were analyzed. Quantification of staining in 5XFAD brain sections is representative of a relative increase in total area or number of distinct foci of intense staining associated with amyloid plaques. Independent areas of intense staining were identified using the FIJI analyze particles tool and are denoted as number of particles per unit area (cm^2^) of brain region to augment our measurements of total area stained as a percentage of total hippocampal or cortical area.

### 2.7. Statistical Analysis

Statistical analysis of NORT and CFR was carried out using ordinary least squares regression to assess the effects of CAW treatment across the collection of three dose treatments and vehicle control. A multi-way ANOVA framework was used to evaluate effects across all treated animals, with specific contrasts of dose treatment done for both genotype (5XFAD vs WT B6/SJL F1 background) and gender, including their interaction. In instances where dose effects on subgroups of gender and genotype were not observed, those interactions were removed, although the main effects were retained, to evaluate the impact of CAW without a loss of power due to limited subgroup sample sizes. CAW dose was primarily considered as a 4-level factor, with vehicle as the reference control, to explicitly identify specific doses that were associated with statistically significant effects and identify minimum dosing levels. Effect sizes are reported as test statistics from the regression models with F-statistics for overall factor effects and t-statistics for individual contrasts. Outcomes presenting with an extensive right skew, such as gene expression and pathology counts, were log-transformed as necessary for modelling purposes. Due to the numerous outcomes and evaluated models, significance was globally defined as *p* < 0.01 to account for multiplicity, while *p* < 0.05 was merely considered a trend. Analyses were performed using R 3.4.4(R Foundation for Statistical Computing, Vienna, Austria).

## 3. Results

### 3.1. CAW Improves Memory in a Dose-Dependent Manner Regardless of Gender or Pathology

**Figure 1:**
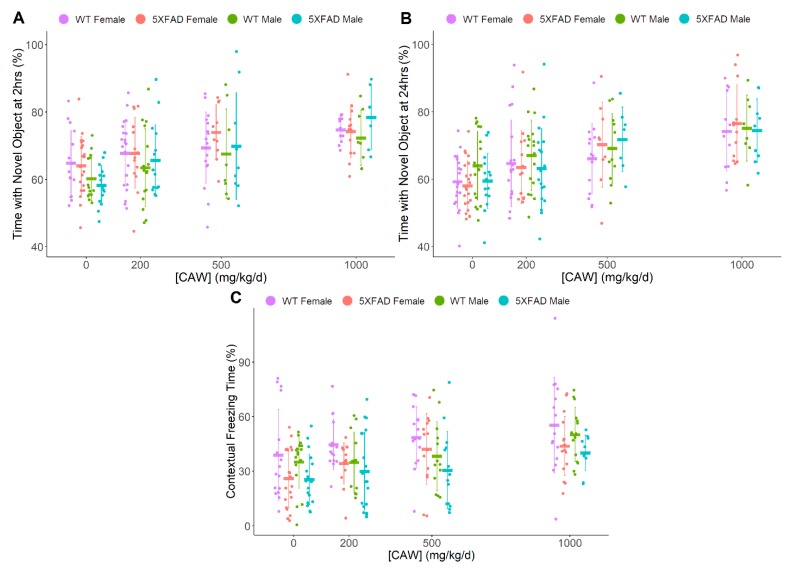
CAW impact on object recall and contextual fear memory. (**A**,**B**) Novel object recognition testing after 3 weeks CAW treatment (A—2 h post-habituation, B—24 h post-habituation) (Combined dose response—2 h *p* < 0.001, 24 h *p* < 0.001, WT vs 5XFAD – 2 h *p* = 0.45, 24 h *p* = 0.36, Females vs Males—2 h *p* = 0.022, 24 h *p* = 0.16) (**C**) Conditioned fear response analysis after 4 weeks CAW treatment (Combined dose response—*p* < 0.001, WT vs 5XFAD—*p* < 0.001, Females vs Males—*p* = 0.011). Individual data points shown with group means and standard deviations. n values: (A/B/C) WT females 0 mg/kg/d n = 15/19/16, 200 mg/kg/d n = 21/21/15, 500 mg/kg/d n = 17/14/15, 1000 mg/kg/d n = 11/12/15; 5XFAD females 0 mg/kg/d n = 21/23/20, 200 mg/kg/d n = 14/14/14, 500 mg/kg/d n = 10/9/15, 1000 mg/kg/d n = 13/12/16; WT males 0 mg/kg/d n = 14/18/17, 200 mg/kg/d n = 16/17/12, 500 mg/kg/d n = 8/11/13, 1000 mg/kg/d n = 8/8/15; 5XFAD males 0 mg/kg/d n = 15/16/18, 200 mg/kg/d n = 15/19/19, 500 mg/kg/d n = 9/7/14, 1000 mg/kg/d n = 6/9/11.

Animal memory was tested via the novel object recognition test (NORT) (Figure 1A,B) and conditioned fear response (CFR) (Figure 1C), respectively. A dose-dependent response to CAW treatment promoting a gain in memory function was observed for all testing paradigms, with a sizable effect in short-term 2 h NORT (F = 15.0, *p* < 0.001), NORT at 24 h post habituation (F = 21.2, *p* < 0.001), as well as CFR (F = 9.12, *p* < 0.001). Gender difference was noted as a non-significant trend, where males performed slightly worse than females on 2 h NORT (F = 5.34, *p* = 0.022) and CFR (F = 6.51, *p* = 0.011), although this was not observed at the 24 h NORT time point. While not seen during NORT testing, the pathology of the 5XFAD mice significantly impaired CFR performance regardless of CAW dose (F = 17.4, *p* < 0.001). However, neither gender nor pathology were observed to modulate the effects of CAW on memory. Significant improvements in the performance of combined groups (i.e., all genders and genotypes) were found at all doses of CAW during NORT evaluation at both the 2 h (200 mg/kg/d t = 2.61, *p* = 0.0096; 500 mg/kg/d t = 4.30, *p* < 0.001; 1000 mg/kg/d t = 6.35, *p* < 0.001) and 24 h time points (200 mg/kg/d t = 2.79, *p* = 0.0057; 500 mg/kg/d t = 4.45, *p* < 0.001; 1000 mg/kg/d t = 7.68, *p* < 0.001) compared to vehicle control. Meanwhile, significant improvement in CFR performance compared to vehicle control was observed at the higher doses of 500 mg/kg/d (t = 2.80, *p* = 0.0056) and 1000 mg/kg/d (t = 5.10, *p* < 0.001).

### 3.2. CAW Has Marginal Benefit to Synaptic Density in Hippocampus of Male Mice

**Figure 2:**
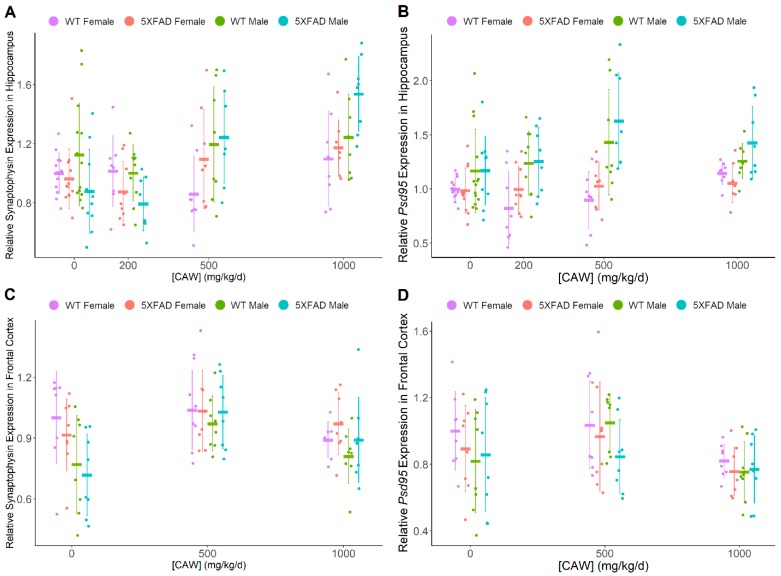
*Centella asiatica* water extract (CAW) impact on markers of synaptic density in the hippocampus and prefrontal cortex. Quantitative PCR analysis of (**A**,**C**) pre-synaptic marker synaptophysin (Combined dose response—hippocampus *p* < 0.001, cortex *p* = 0.001; WT vs 5XFAD—hippocampus *p* = 0.81, cortex *p* = 0.75; Females vs. Males—hippocampus *p* = 0.016, cortex *p* = 0.004) and (**B**,**D**) post-synaptic marker *Psd95* (Combined dose response—hippocampus *p* = 0.026, cortex *p* = 0.005; WT vs 5XFAD-hippocampus *p* = 0.17, cortex *p* = 0.18; Females vs. Males—hippocampus *p* < 0.001, cortex *p* = 0.19) in the (**A**,**B**) hippocampus and (**C**,**D**) frontal cortex of nine month old WT and 5XFAD male and female littermates. All groups are expressed relative to female WT untreated control. Individual data points shown with group means and standard deviations. n values: (A/B/C/D) WT females 0 mg/kg/d n = 13/13/8/8, 200 mg/kg/d n = 8/8/0/0, 500 mg/kg/d n = 8/8/8/8, 1000 mg/kg/d n = 8/8/7/8; 5XFAD females 0 mg/kg/d n = 12/12/8/8, 200 mg/kg/d n = 9/9/0/0, 500 mg/kg/d n = 8/8/8/8, 1000 mg/kg/d n = 8/8/8/8; WT males 0 mg/kg/d n = 14/14/8/8, 200 mg/kg/d n = 10/10/0/0, 500 mg/kg/d n = 8/8/8/8, 1000 mg/kg/d n = 8/8/8/8; 5XFAD males 0 mg/kg/d n = 10/10/8/8, 200 mg/kg/d n = 8/8/0/0, 500 mg/kg/d n = 8/8/8/8, 1000 mg/kg/d n = 8/8/8/8.

Transcript expression of presynaptic marker synaptophysin and post-synaptic marker *Psd95* measured via quantitative PCR was used as an indicator of synaptic density (Figure 2). An inconsistent CAW dose-response effect was observed across all groups for *Psd95* in both hippocampus and frontal cortex, primarily driven by the male 5XFAD group. There was not a significant effect of pathology on synaptophysin or *Psd95* in the hippocampus or cortex or an effect of gender on these synaptic markers in the hippocampus. Male mice showed reduced gene expression of cortical synaptophysin vs females (F = 8.90, *p* = 0.004). Table 1 provides a summary of this transcript expression data. The elevation of hippocampal synaptophysin expression with CAW treatment in general, was moderated by pathology in 5XFAD mice vs WT CAW-treated mice (F = 3.99, *p* = 0.009) but when individual doses were directly compared to untreated 5XFAD mice, this effect was only found as a non-significant trend at the higher doses (500 mg/kg/d: t = 2.24, *p* = 0.027; 1000 mg/kg/d: t = 2.59, *p* = 0.011). There was also a suggestion of a gender-moderated increase in *Psd95* with CAW treatment in male hippocampi (F = 3.18, *p* = 0.026). The interaction between dose and gender (across genotypes) was not quite significantly increased in the hippocampus at a dose of 500 mg/kg/d when compared to no treatment (t = 2.99, *p* = 0.033) and was lost at the highest dose of 1000 mg/kg/d (t = 0.50, *p* = 0.62). Hippocampal synaptophysin transcript levels were found to be significantly increased by CAW treatment only in 5XFAD male mice at the highest CAW dose of 1000 mg/kg/d vs WT CAW 1000 mg/kg/d male mice (t = 2.86, *p* = 0.006) and vs 5XFAD CAW 1000 mg/kg/d female mice (t = 2.73, *p* = 0.008). There was a suggestion of a CAW-mediated increase of *Psd95* expression in male hippocampi regardless of genotype vs CAW-treated females but was not statistically significant (F = 4.71, *p* = 0.032). 

### 3.3. CAW Increases ARE Gene Expression in Hippocampus Regardless of Gender or Pathology

**Figure 3:**
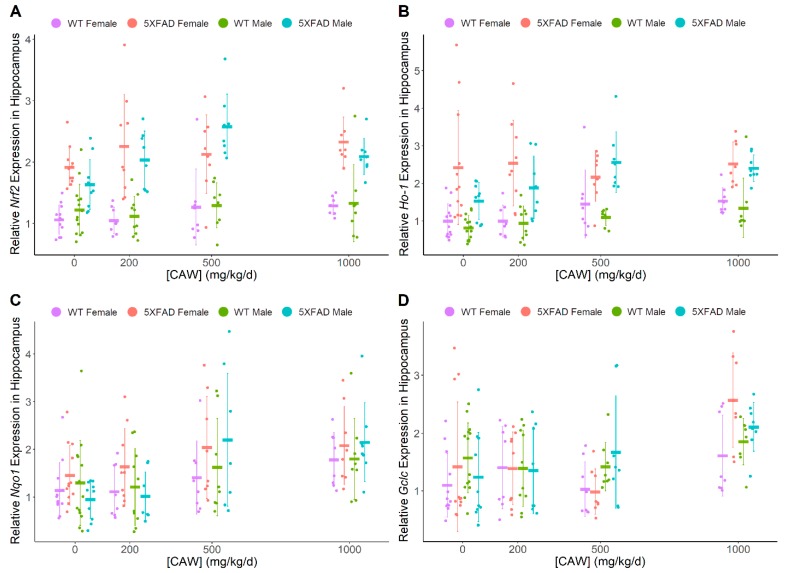
CAW impact on markers of oxidative stress in hippocampus. Quantitative PCR analysis of NRF2-regulated antioxidant genes in the hippocampus of nine month old WT and 5XFAD male and female littermates: (**A**) *Nrf2* (Combined dose response *p* = 0.007; WT vs. 5XFAD *p* < 0.001; Females vs. Males *p* = 0.57) (**B**) *Ho-1* (Combined dose response *p* < 0.001; WT vs. 5XFAD *p* < 0.001; Females vs Males *p* = 0.020) (**C**) *Nqo1* (Combined dose response *p* < 0.001; WT vs. 5XFAD *p* = 0.064; Females vs. Males *p* = 0.85) (**D**) *Gclc* (Combined dose response *p* < 0.001; WT vs. 5XFAD *p* = 0.64; Females vs. Males *p* = 0.066) All groups are expressed relative to female WT untreated control. Due to the heavy skew, all expression levels were logged transformed to better conform to model assumptions of normality. Individual data points shown with group means and standard deviations. n values: (A/B/C/D) WT females 0 mg/kg/d n = 13/13/11/11, 200 mg/kg/d n = 8/8/8/8, 500 mg/kg/d n = 7/7/8/8, 1000 mg/kg/d n = 8/8/8/8; 5XFAD females 0 mg/kg/d n = 12/12/12/11, 200 mg/kg/d n = 9/9/9/9, 500 mg/kg/d n = 8/8/8/8, 1000 mg/kg/d n = 8/8/8/8; WT males 0mg/kg/d n = 14/14/13/14, 200mg/kg/d n = 10/10/10/10, 500 mg/kg/d n = 8/8/8/8, 1000 mg/kg/d n = 8/7/8/8; 5XFAD males 0 mg/kg/d n = 10/9/10/10, 200 mg/kg/d n = 8/8/8/8, 500 mg/kg/d n = 8/7/8/8, 1000 mg/kg/d n = 8/8/8/8.

**Figure 4:**
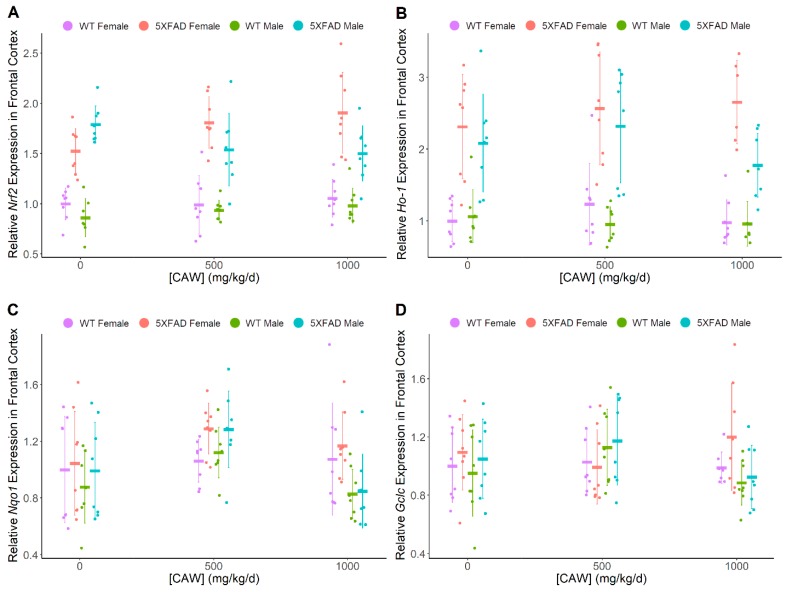
*CAW impact on markers of oxidative stress in prefrontal cortex.* (A–D) Quantitative PCR analysis of NRF2-regulated antioxidant genes in the frontal cortex of nine month old WT and 5XFAD male and female littermates: (**A**) *Nrf2* (Combined dose response *p* = 0.53, WT vs. 5XFAD *p* < 0.001, Females vs. Males *p* = 0.042) (**B**) *Ho-1* (Combined dose response *p* = 0.32, WT vs. 5XFAD *p* < 0.001, Females vs. Males *p* = 0.073) (**C**) *Nqo1* (Combined dose response *p* = 0.001, WT vs. 5XFAD *p* = 0.068, Females vs. Males *p* = 0.042) (**D**) *Gclc* (Combined dose response *p* = 0.43, WT vs. 5XFAD *p* = 0.20, Females vs. Males *p* = 0.46). All groups are expressed relative to female WT untreated control. Individual data points shown with group means and standard deviations. n values: (A/B/C/D) WT females 0 mg/kg/d n = 8/8/8/8, 500 mg/kg/d n = 8/7/8/8, 1000 mg/kg/d n = 8/8/8/7; 5XFAD females 0 mg/kg/d n = 8/8/8/8, 500 mg/kg/d n = 8/8/8/8, 1000 mg/kg/d n = 8/8/8/8; WT males 0 mg/kg/d n = 7/7/7/8, 500 mg/kg/d n = 8/8/8/8, 1000 mg/kg/d n = 7/7/8/8; 5XFAD males 0 mg/kg/d n = 8/8/8/8, 500 mg/kg/d n = 8/8/8/8, 1000 mg/kg/d n = 8/8/7/8.

CAW was found to have a dose-dependent impact on levels of hippocampal ARE genes of interest (Figure 3), including *Nrf2* (F = 4.22, *p* = 0.007), *Ho-1* (F = 7.84, *p* < 0.001), *Nqo1* (F = 9.29, *p* < 0.001), and *Gclc* (F = 8.76, *p* < 0.001), irrespective of gender or genotype. 5XFAD mice exhibited higher expression levels of *Nrf2* (hippocampus F = 137, *p* < 0.001, frontal cortex F = 162.3, *p* < 0.001) and *Ho-1* (hippocampus F = 112, *p* < 0.001, frontal cortex F = 94.0, *p* < 0.001) than WT littermates. Gender was not significantly associated with differences in antioxidant gene expression in either hippocampus or frontal cortex. Neither pathology nor gender influenced the relationship between hippocampal gene expression and CAW treatment. When directly compared to untreated controls, the highest dose of 1000 mg/kg/d significantly increased expression for all four genes (*Nrf2*: t = 2.66, *p* = 0.009; *Ho-1*: t = 4.38, *p* < 0.001; *Nqo1*: t = 4.43, *p* < 0.001; *Gclc*: t = 4.57, *p* < 0.001) while the middle dose of 500 mg/kg/d increased expression for every gene except *Gclc* (*Nrf2*: t = 3.20, *p* = 0.002; *Ho-1*: t = 3.22, *p* = 0.002; *Nqo1*: t = 3.06, *p* = 0.003) in hippocampal samples. The lowest dose of 200 mg/kg/d did not increase hippocampal mRNA expression for any of the antioxidant genes. Changes in cortical ARE gene expression levels were less consistent than those in the hippocampus (Figure 4). CAW treatment in 5XFAD mice showed a gender disparity, where female mice exhibited non-significant trends with increased cortical levels of *Nrf2* at 500 (t = 1.93, *p* = 0.061) and 1000 mg/kg/d (t = 2.59, *p* = 0.013) while 1000 mg/kg/d CAW-treated male mice showed a significant decrease relative to untreated controls (t = −3.23, *p* = 0.002, 500 mg/kg/d vs control t = −2.57, *p* = 0.013). Although increased cortical expression of *Nqo1* was observed at 500 mg/kg/d in mice regardless of gender or genotype (t = 3.44, *p* < 0.001), this effect was not observed at a dose of 1000 mg/kg/d. Table 1 provides a summary of transcript expression data. 

### 3.4. CAW Does not Alter Amyloid-β Plaque Burden

**Figure 5:**
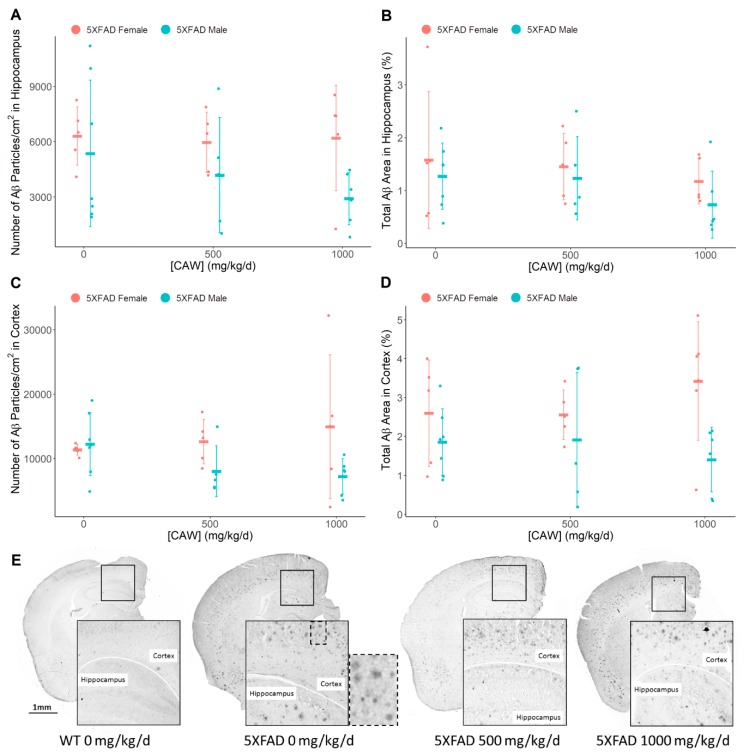
CAW impact on 5XFAD plaque burden. Pan Aβ immunohistochemical analysis of number of particles and total plaque area within the (**A**,**B**) hippocampus (Number of Aβ particles *p* = 0.74, Total Aβ area *p* = 0.86), and (**C**,**D**) cortex (Number of Aβ particles *p* = 0.66, Total Aβ area *p* = 0.93) of 5XFAD male and female littermates. Individual data points shown with group means and standard deviations. (**E**) Images of pan Aβ-stained coronal sections of right hemisphere. Dashed line box of untreated 5XFAD Aβ IHC expanded to show staining of amyloid plaques. n values: (A/B/C/D) 5XFAD females 0 mg/kg/d n = 5/5/5/5, 500 mg/kg/d n = 5/5/5/5, 1000 mg/kg/d n = 5/5/6/6; 5XFAD males 0 mg/kg/d n = 7/7/7/7, 500 mg/kg/d n = 5/5/5/5, 1000 mg/kg/d n = 6/6/6/6.

IHC analysis of pan-Aβ staining within the cortical and hippocampal regions of nine month old 5XFAD mice did not indicate changes in the amyloid-β plaque burden due to CAW treatment (Figure 5) in either Aβ particle count (cortex: F = 0.41, *p* = 0.66; hippocampus: F = 0.30, *p* = 0.74) or in total plaque area (cortex: F = 0.07, *p* = 0.93; hippocampus: F = 0.14, *p* = 0.86). Male mice (all combined treatments: 0, 500, 1000 mg/kg/d CAW) were observed to have reduced plaque area in the cortex compared to all female mice (F = 7.90, *p* = 0.009).

### 3.5. CAW Reduces Aβ Plaque-Associated Oxidative Stress in both the Cortex and the Hippocampus

**Figure 6:**
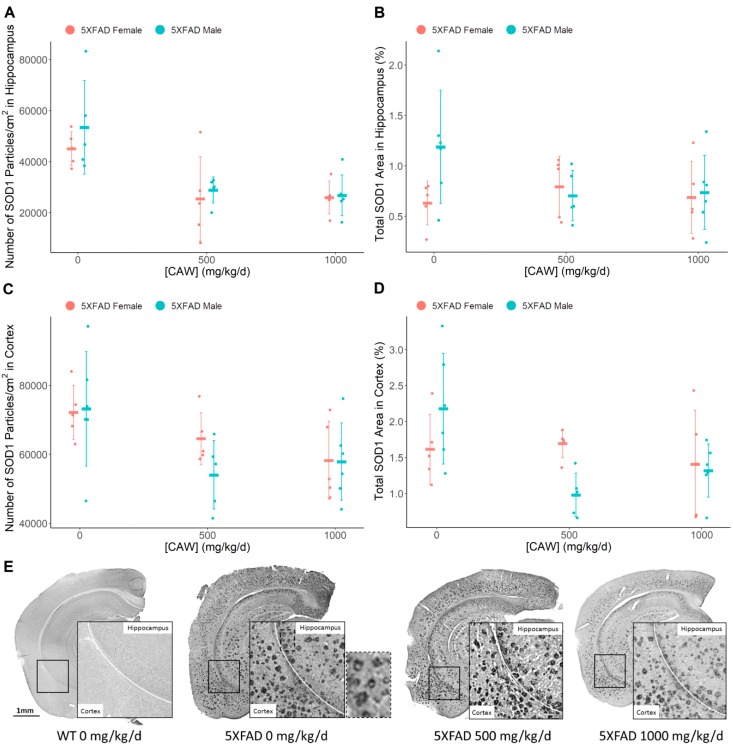
CAW impact on Aβ-associated oxidative stress in the hippocampus and cortex of 5XFAD mice. SOD1 immunohistochemical analysis of number of particles and total area within the (**A**,**B**) hippocampus (Number of SOD1 particles *p* < 0.001, Total SOD1 area *p* = 0.38), and (**C**,**D**) cortex (Number of SOD1 particles *p* = 0.008, Total SOD1 area *p* = 0.042) of 5XFAD male and female littermates. Individual data points shown with group means and standard deviations. (**E**) Images of SOD1-stained coronal sections of right hemisphere. Dashed line box of untreated 5XFAD expanded to show SOD1 IHC staining presumed to be around amyloid plaques seen in Figure 5E. n values: (A/B/C/D) 5XFAD females 0 mg/kg/d n = 5/5/5/5, 500 mg/kg/d n = 5/5/5/5, 1000 mg/kg/d n = 5/5/5/5; 5XFAD males 0 mg/kg/d n = 6/6/6/6, 500 mg/kg/d n = 5/5/5/5, 1000 mg/kg/d n = 6/6/6/6.

Based on published reports and our experience, IHC for SOD1 labels areas of high oxidative stress, specifically around Aβ plaques, and is associated with dystrophic neurites [21,48]. IHC evaluation of superoxide dismutase 1 (SOD1) illustrated a significant reduction in enzyme particle number per square centimeter in both the hippocampus (F = 15.5, *p* < 0.001) and the cortex (F = 5.67, *p* = 0.008) (Figure 6) following CAW treatment of male and female 5XFAD mice. Meanwhile, only a non-significant trend in a reduction of total SOD1 area was observed in the cortex (F = 3.55, *p* = 0.042) with no effect observed in the hippocampus (F = 1.01, *p* = 0.38). Compared to untreated controls, a significant reduction in SOD1 particles was seen at both doses in the hippocampus (combined genders: 500 mg/kg/d: t = −4.63, *p* < 0.001; 1000 mg/kg/d: t = −4.94, *p* < 0.001); however, a reduction in cortical SOD1 particle number was only observed at the higher dose of 1000 mg/kg/d dose (t = −3.041, *p* = 0.005) with a non-significant trend at 500 mg/kg/d (t = 2.75, *p* = 0.010).

## 4. Discussion

We utilized 5XFAD mice in this study to validate CAW’s benefit to cognitive function and elucidate its effect on oxidative stress and Aβ plaque formation in vivo. The 5XFAD model allows for an accelerated timetable of cognitive dysfunction associated with Aβ-related AD pathology in comparison to the Tg2576 model [34,35] which was used in our prior studies. 5XFAD mice show impaired remote memory stabilization prior to four months of age [49]; impaired spatial memory in the Y-maze at 4–5 months of age [35], Morris water maze at six months of age [50,51], and radial water maze at eight months of age [52]; decreased working memory and learning during NORT testing at six months of age [50]; and decreased contextual fear memory by six months of age [38,49,53]. We tested object recall (NORT) and contextual fear (CFR) memory of approximately 8–9 month old female and male 5XFAD mice and WT littermates treated with CAW for 3–4 weeks. The use of both genders in the 5XFAD model allows for analyses, relevant to the gender-related differences in AD patients. 

Previous studies in our laboratory have shown CAW treatment (single dose of 200 mg/kg/d for four weeks) of 20 month old Tg2576 female mice improves spatial memory and learning (as measured by Morris water maze) without impacting Aβ_1-40_ or Aβ_1-42_ levels in the cortex [13]. This treatment benefit to memory was recapitulated in the present study which examined approximately 8–9 month old 5XFAD mice of both genders treated with a range of CAW doses (200, 500, or 1000 mg/kg/d for 3–4 weeks). Here, CAW improved working and contextual fear memory, again without impacting Aβ plaque burden. CAW treatment (3–4 weeks) demonstrated memory improvement in both the NORT (200–1000 mg/kg/d) and CFR (500–1000 mg/kg/d) evaluations. A pathology-dependent memory deficit during CFR testing was observed as expected, but was not seen upon NORT evaluation. During CFR, a trending elevated baseline fear memory in female mice versus males was also observed. A fear-mediated relative memory deficit in males during CFR testing is not unexpected and validates previous CFR testing in mice [54,55,56,57].

CAW dose-dependent effects on behavior were not modulated by pathology or gender. A treatment benefit of CAW (200 mg/kg/d for four weeks) in WT animals was previously reported both in male and female aged (20 month old) WT mice (as measured by Morris water maze, object location, and novel object recognition) [1,46]. This was recapitulated by CAW dose-dependent (200–1000 mg/kg/d for 3–4 weeks) cognitive benefits to both male and female 7–9 month old WT mice in the present study.

To elucidate the mechanisms underlying CAW’s memory benefit, we analyzed changes in the health of hippocampal neurons via mRNA expression of synaptophysin and *Psd95*, markers of pre-synaptic and post-synaptic density, respectively [58,59,60,61]. We found a statistically significant CAW treatment-dependent increase in these synaptic markers in the cortex, while there was a significant increase in synaptophysin in the hippocampus and an increasing trend in *Psd95* (*p*=0.026) with CAW treatment. An interesting observation in the progression of 5XFAD pathology is that spine loss (4–9 months) [62] occurs subsequent to cognitive dysfunction (3–6 months) [35,63,64,65,66] and Aβ plaque formation (two months) [67]. This is different from the progression in Tg2576, which manifests synaptic damage at an early stage of pathological development (4.5 months) [68,69] with a corresponding decrease in synaptic density markers [70] prior to Aβ plaque development (10 months) [71] and cognitive deficiencies (one year) [72,73]. While previous studies have shown minimal hippocampal synaptic damage in 5XFAD mice [62], the positive impact of CAW on synaptic formation in the hippocampus (or other brain regions) may not require this pathological deficit to be present, to improve neuronal function and provide cognitive benefits.

Our observations regarding these synaptic markers also elicited suggestions of gender differences in response to CAW treatment. We observed increased expression of synaptophysin in the cortices of female mice and higher expression of hippocampal *Psd95* in males. In addition, we observed elevated hippocampal synaptophysin in 5XFAD males vs. 5XFAD females treated with 1000 mg/kg/d CAW (*p* = 0.008), as well as a trend of an increase in *Psd95* in 500 mg/kg/d CAW-treated male mice vs. females, regardless of genotype (*p* = 0.032). These data are inconclusive but suggest a possible gender-specific impact on synaptic density in the hippocampus after CAW treatment, which requires further study. Indications of decreased synaptophysin in untreated 5XFAD mice vs. untreated WT mice (*p* = 0.063), suggesting poorer synaptic density due to pathology. are consistent with previous studies of nine month old 5XFAD mice [35]. Furthermore, a consistent decrease in synaptophysin or PSD95 has not been observed prior to nine months of age [35,63,74,75,76]. Future experiments with mice over nine months of age might exacerbate this difference (AD pathology due to Aβ toxicity is age-dependent), potentially leading to more robust synaptic pathology, allowing for a more effective evaluation of CAW’s impact on synaptic density. It would also be advantageous in future experiments to utilize a larger set of synaptic markers, such as synapsin I, VGLUT1, or CaMKIIb [77,78], whole transcriptome analyses, direct measurement of fluorescently labeled dystrophic neurites [79] or other analysis of protein markers of neuritic dystrophy [80,81].

Oxidative stress associated with Aβ pathology plays an important role in the death of neurons in the AD brain [82,83,84,85]. The NRF2 antioxidant signaling pathway (as well as several other mediators) serves as part of the cellular defense against ROS-mediated oxidative damage during normal mitochondrial function and as a result of disease pathology. Previous studies in our laboratory show that CAW treatment is associated with elevated levels of NRF2-targeted ARE gene transcripts in Tg2576 primary neurons [27], as well as the hippocampus and cortex of young and aged WT mice [46]. The positive association of CAW dose response with these ARE gene transcripts (*Nrf2*, *Ho-1*, *Nqo1*, *Gclc*) in the hippocampus in these studies provides further evidence for the antioxidant activity inherent in CAW treatment. This would potentially facilitate the protection of hippocampal neurons, the mechanisms of which are currently under study. Numerous other studies demonstrate both an increase and decrease in ARE gene expression in AD brain tissue, citing an initial heightened antioxidant response due to oxidative stress [22,86,87], followed by its eventual exhaustion with the decrease of ARE pathway signaling [88]. This transient response makes it difficult to determine the antioxidant mechanisms of CAW in concert with the response to oxidative stress due to AD pathology without first analyzing a time course of oxidative stress and subsequent response in the 5XFAD model. In the 8–9 month old mice tested here, *Nrf2* and *Ho-1* were both upregulated in 5XFAD vs. WT mice. These NRF2 targets, and others, are known to be induced by multiple compounds found in CA. Compounds such as the phenolic ferulic acid [89,90] and the triterpene asiatic acid [2,91], induce *Nrf2* and its ARE-regulated targets, exhibiting a neuroprotective effect [92,93,94,95]. Caffeoylquinic acids found in CA [28,96,97], are also known to induce *Nrf2* and *Ho-1* signaling, and positively impact glutathione levels [98,99].

In the current study, we observed an increase in total ARE gene expression in the hippocampus, and memory improvement in CAW-treated 5XFAD mice, without an impact on Aβ plaques. This suggests a mitigating impact on oxidative stress regardless of plaque burden in these animals. However, in our previous experiments, CAW treatment has been seen to reduce Aβ plaques in 5XFAD hippocampus [33] where a higher baseline Aβ burden (> 3%) was present. The impact of CAW on 5XFAD Aβ plaque burden should be resolved in future experiments. Data from other CA extract-treated AD mouse models are similarly inconsistent. Our previous studies in aged female Tg2576 mice treated with CAW also showed no impact on cortical Aβ_40_ or Aβ_42_ levels but did not test for a possible effect on plaque aggregation [13]. However, a study in a PSAPP mouse model of AD has shown a reduction of Aβ following treatment with an ethanolic extract of CA (which would have a different chemical profile to CAW) [12] when administered at a higher dose for a longer duration of time (2500–5000 mg/kg/d for 2–8 months). These reports highlight the need for further exploration into CA’s impact on amyloid plaque burden.

Our present data in the 5XFAD model is congruent with other literature reports in both mice and humans with AD pathology. For instance, Hong et al. (2013) used proteomic analysis to demonstrate activation of the NRF2 transcriptional pathway in 5XFAD mice at four months of age [100], which is consistent with ARE activation in Alzheimer’s patients [101]. Our laboratory has shown *Nrf2* expression, specifically, is elevated further with CAW treatment in seven month old 5XFAD female mice [33], an observation shown in the present study to occur in a CAW dose-dependent manner. This suggests CAW improves antioxidant signaling to combat the ever increasing oxidative stress mediated by the expanding Aβ pathology indicative of AD. Supplementation of NRF2 antioxidative mechanisms using the NRF2 activator sulforaphane improves cognitive function in 5XFAD and 3xTg-AD mouse models [102], providing supporting evidence of the cognitive benefit of added *Nrf2* expression beyond that of the native 5XFAD pathology-driven increase. 

**Figure 7:**
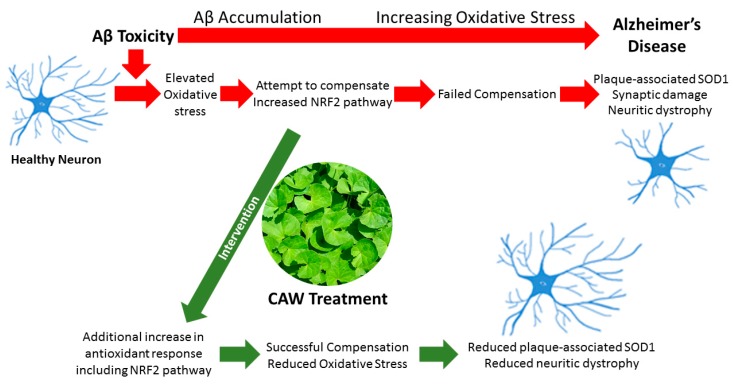
Putative model for CAW antioxidative protection of 5XFAD neurons. CAW facilitates increased expression of NRF2-mediated ARE gene expression in the hippocampus to heighten antioxidant response to Aβ oxidative toxicity. CAW-mediated mitigation of increased ROS in neurons, results in reduced oxidative stress and, subsequently, plaque-associated SOD1, which correlates with reduced neuritic dystrophy, and ultimately prevents permanent synaptic damage and neuronal death.

NRF2 pathway signaling is part of a larger cellular response to oxidative stress, a response which includes the key regulator of ROS content, superoxide dismutases. Griñán-Ferré et al. (2016) showed SOD1 protein is elevated in the hippocampi of 5XFAD mice vs WT controls at eight months of age [103]. Elevated SOD expression is indicative of the high levels of oxidative stress associated with Aβ plaques in Alzheimer’s disease brains [21,48,80,104]. Our IHC analysis revealed that CAW treatment was associated with a reduction in Aβ plaque-associated oxidative stress in the hippocampus and cortex of 5XFAD mice. We interpret the improvement in behavior to mean that CAW provides a neuroprotective effect against Aβ toxicity through the attenuation of oxidative stress and possible mitigation of neuritic dystrophy around the plaques, as the brain Aβ load is unchanged by treatment (Figure 7). This is supported by previous research, which utilized antioxidant therapies to reduce oxidative stress and demonstrated the reversible nature of the neuritic dystrophy observed due to AD pathology in APP/PS1 transgenic mice [105]. Therefore, we postulate that CAW mitigation of oxidative stress is at least in part through ARE antioxidant signaling, as demonstrated by reduced Aβ-associated SOD1 levels. 

The memory benefits of CAW observed in this study and others may be due to the complex interactions of multiple mechanisms, in addition to the promotion of an antioxidant response illustrated here. This is suggested by the cognitive effects observed in WT mice, where only baseline levels of oxidative stress would be expected to be present. We have previously observed increased hippocampal and cortical mitochondrial gene expression [46] and increased mitochondrial activity [33] in response to CAW treatment. To better encapsulate the multifaceted impact of whole plant extract treatment, we are continuing analyses of brain tissue using untargeted metabolomics to elucidate key mediators of CAW activity. 

Numerous active CAW compounds are currently being examined in our laboratory to elucidate their effects on cognitive function, oxidative stress, and mitochondrial biogenesis and function. Previously, in vitro studies have shown that both CAW whole extract and isolated compounds (detailed in a review [32]) facilitate neurite outgrowth [6,28,106], and elicit neuroprotection [107,108,109,110,111]. The neurological effects of certain CAW active compounds are also observed in vivo, increasing learning and memory while providing neuroprotection in rodents [2,13,112]. The effects of some of these active compounds (specifically triterpenes and caffeoylquinic acids) are currently being evaluated in 5XFAD mice in our laboratory. These multifaceted aspects of CAW activity require further study to facilitate integration with the full scale of AD pathology. The in vivo models we use in our laboratory (Tg2576, 5XFAD) do not possess tau tangles (an important part of AD pathology) although we have implemented cell culture models which show CAW’s ability to mitigate tau expression and phosphorylation [113]. In addition, we have yet to analyze the impact of CAW on other important mechanisms in AD, including ER stress, calcium dys-homeostasis, mitochondrial dysfunction, and downstream oxidative stress. Many of these areas of focus are currently under study. However, the evidence is sufficient to begin to consider preliminary clinical trials of CAW, and the studies described here have been important for refining the dose and outcome measures for early phase studies. Our group has recently initiated clinical studies (ClinicalTrials.gov Identifiers NCT03929250—Pharmacokinetics of *Centella asiatica* in the Elderly, and NCT03937908—Pharmacokinetics *Centella asiatica* Product (CAP) in Mild Cognitive Impairment, supported by NIH grant numbers R61AT009628 and P30AG008017) which will proceed in parallel with mechanistic studies in animal models. 

## 5. Conclusions

Treatment of AD mouse model 5XFAD and WT littermates with a water extract of *Centella asiatica* (CAW) showed a dose-dependent improvement in memory regardless of gender or pathology, without impacting the amyloid plaque burden. The memory improvement with CAW treatment may be attributed to an increase in ARE gene expression, primarily in the hippocampus, indicating a putative reduction in oxidative stress in addition to the natural cellular response to pathology. Furthermore, CAW treatment reduced Aβ plaque-associated SOD1 in the hippocampus and cortex. Therefore, we postulate that CAW treatment promotes an antioxidative response in opposition to increased oxidative stress driven by Aβ pathology, which mitigates neuritic dystrophy in neurons surrounding the amyloid plaques that ultimately maintains cognitive function. 

## Figures and Tables

**Table 1:** Summary of CAW impact on target mRNA in hippocampus and prefrontal cortex. Significant gene expression changes observed in quantitative PCR analysis of synaptic and NRF2-regulated antioxidant genes in the hippocampus and frontal cortex of nine month old WT and 5XFAD male and female littermates.

Gene	Dose Response Effect: CAW vs Control Across Genders and Genotypes		Genotype Effect: 5XFAD vs WT Across Genders and Treatments		Gender Effect: Females vs Males Across Genotypes and Treatments	
	Hippocampus	Cortex	Hippocampus	Cortex	Hippocampus	Cortex
**Synaptophysin**	↑	↑	(-)	(-)	(-)	F > M
***Psd95***	(-)	↑	(-)	(-)	M > F	(-)
***Nrf2***	↑	(-)	5XFAD > WT	5XFAD > WT	(-)	(-)
***Ho-1***	↑	(-)	5XFAD > WT	5XFAD > WT	(-)	(-)
***Nqo1***	↑	↑	(-)	(-)	(-)	(-)
***Gclc***	↑	(-)	(-)	(-)	(-)	(-)

(-) no significant effect (*p* > 0.01); ↑ significantly up-regulated (*p* < 0.01); ↓ significantly down-regulated (*p* < 0.01); M—Male; F—Female; WT—Wild Type.
